# Research on a Mixed Gas Recognition and Concentration Detection Algorithm Based on a Metal Oxide Semiconductor Olfactory System Sensor Array

**DOI:** 10.3390/s18103264

**Published:** 2018-09-28

**Authors:** Yonghui Xu, Xi Zhao, Yinsheng Chen, Wenjie Zhao

**Affiliations:** 1 School of Electrical Engineering and Automation, Harbin Institute of Technology, Harbin 150001, China; xyh@hit.edu.cn (Y.X.); richard214@163.com (X.Z.); 2 School of Measurement and Control Technology and Communication Engineering, Harbin University of Science and Technology, Harbin 150001, China; zwjsky888@163.com

**Keywords:** sensor array, gas detection, gas identification, kernel principal component analysis, multivariate relevance vector machine

## Abstract

As a typical machine olfactory system index, the accuracy of hybrid gas identification and concentration detection is low. This paper proposes a novel hybrid gas identification and concentration detection method. In this method, Kernel Principal Component Analysis (KPCA) is employed to extract the nonlinear mixed gas characteristics of different components, and then K-nearest neighbour algorithm (KNN) classification modelling is utilized to realize the recognition of the target gas. In addition, this method adopts a multivariable relevance vector machine (MVRVM) to regress the multi-input nonlinear signal to realize the detection of the concentration of the hybrid gas. The proposed method is validated by using CO and CH_4_ as the experimental system samples. The experimental results illustrate that the accuracy of the proposed method reaches 98.33%, which is 5.83% and 14.16% higher than that of principal component analysis (PCA) and independent component analysis (ICA), respectively. For the hybrid gas concentration detection method, the CO and CH_4_ concentration detection average relative errors are reduced to 5.58% and 5.38%, respectively.

## 1. Introduction

With the rapid development of modern science and technology, sensor technology and pattern recognition methods continue to improve, promoting the development of machine olfaction. Machine olfaction is a bionic detection technology that uses electronic devices to simulate biological olfactory systems. The system is widely used in gas/odour qualitative identification and quantitative detection. Unlike machine vision technology, which shows mature development and wide application, machine olfaction technology is still in the stage of research and development. This technology shows a wide range of application prospects in such fields as environmental monitoring [1], medical auxiliary diagnosis [2,3], industrial production [4], and public safety [5]. Relevant scholars have continuously devoted themselves to the research of related technologies (gas-sensitive materials, manufacturing processes, signal processing methods, pattern recognition methods, gas molecular structures, etc.) in machine olfaction and have obtained a series of research results [6,7,8]. Mammals can use their natural olfactory system to easily identify some odors, but they cannot easily detect certain colorless, odorless gases [9]. In particular, when certain flammable and explosive toxic gases (such as carbon monoxide and methane) are leaked, they will cause harm at a certain concentration, which will greatly endanger human life and property. Therefore, it is of great significance to qualitatively identify and quantitatively detect various hazards and harmful gases in human production and living environments.

**Figure 1:**
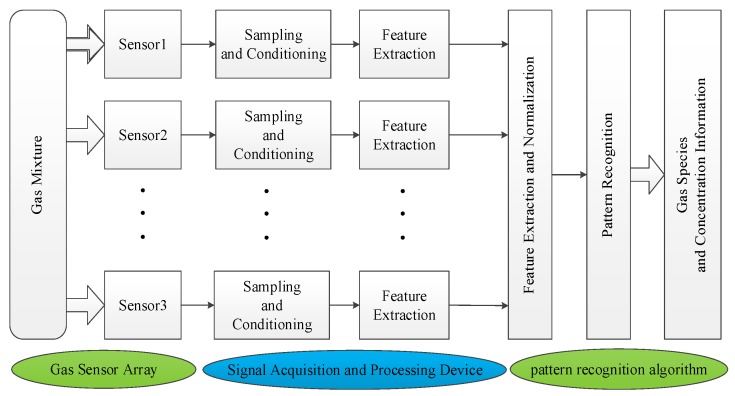
Block diagram of a machine olfactory system.

The machine olfactory system includes three main components: (1) a gas sensor array; (2) a signal acquisition and processing device; (3) a pattern recognition algorithm, as shown in Figure 1. Gas sensor collects the electrical signals, and the combination of various sensors can effectively improve the selection ability of the sensor [10]. The electrical signal output from the sensor passes through the data acquisition and A/D conversion system to obtain a series of response signals. After pre-processing techniques, feature parameters are extracted from each response signal, and the feature values are then extracted through multi-dimensional signal processing techniques. Finally, the feature parameters or feature values are sent to a pattern recognition system to obtain information pertaining to gas type and concentration. The pattern recognition algorithm is composed of two main parts: qualitative identification and quantitative detection. The operating requirements of gas sensors, as key devices for gas detection, have higher and higher requirements for its accuracy, performance and stability [11]. The performance indicators of gas sensors mainly include sensitivity, selectivity, response time, energy consumption, reversibility, adsorption capacity, and fabrication cost. However, the occurrence of certain factors will lead to instability of the gas sensor, such as structural changes, phase shifts, and changes in the surrounding environment. Therefore, to ensure that the gas sensor produces a stable and reproducible signal over a given period, the use of chemically and thermally stable materials to optimize the sensing element’s elemental composition and grain size is critical [12,13]. Among many types of sensors, metal oxide semiconductor (MOS) gas sensors are widely used in gas detection due to their fast response, low cost, and long service life [14,15]. However, because the cross-sensitivity characteristic is prevalent in MOS gas sensors and there is no single-gas selective gas sensor, the use of a single MOS gas sensor cannot recognize a mixed gas [16,17]. In machine olfactory systems, MOS gas sensor array technology is widely used. The basic structure is to form a sensor array for a group of MOS gas sensors with different gas sensitivities. This method improves the selectivity of a single gas sensor [18] and obtains more information on multi-channel response signals, providing a feasible means for the detection and analysis of the composition of mixed gases [19].

Traditional machine olfactory systems rely on MOS gas sensor array technology and pattern recognition algorithms to detect and analyse mixed gases [20,21]. The current research direction of machine olfaction is mainly the development of MOS gas-sensitive materials and the study of pattern recognition algorithms [22]. An effective signal acquisition device is the premise of the machine olfactory system. The selectivity and sensitivity of the MOS gas sensor can be improved through composite materials, preparation processes and doping methods [23]. However, there is no single selective material for the study of MOS gas sensitivity, and cross-sensitive characteristics still exist. The improvement of the detection and analysis performance of the gas mixture components by means of gas-sensitive materials alone does not yield satisfactory results. Hence, many studies have focused on the use of signal processing methods to improve the detection and analysis performance of the machine olfactory system. Some studies [24,25] have noted the importance of the signal processing method in the machine olfactory system. However, improving the performance of the algorithm can better achieve a signal classification effect and enhance the detection and analysis capabilities of the machine olfactory system. One study [26] used a chaotic BPNN algorithm to identify distilled liquors; the recognition rate reached 100%, and the convergence speed was 75.5 times faster that of the BPNN algorithm. Zhang used the LPC pattern recognition algorithm based on KPCA to enhance the elimination of background interference and improved the prediction accuracy of mixed gases [27,28].

Pattern recognition can be defined as the identification or classification of complex signal samples. The current machine olfactory system mainly includes two parts: gas qualitative recognition and quantitative detection [29]. Therefore, the machine olfactory system can also be defined as a pattern recognition. In [30], principal component analysis (PCA) and an artificial neural network (ANN) were used to combine the optimum feature parameters. Using PCA, good separation between the mixed gas signals was obtained, and the recognition probability of the artificial neural network was 98%. Wang [31] proposed a DQN active perception strategy with a higher classification accuracy than that of PCA, which can optimize the flow modulation online, achieve higher recognition accuracy, increase recognition speed, and reduce training and testing costs. Studies [32,33,34] used independent component analysis (ICA) to decompose a multidimensional vector into statistical components, which were as independent as possible and eliminated the redundancy of the original data. In [35], researchers compared the MLP gas quantitative detection performance of single multiple inputs multiple outputs (SMIMO) and multiple multiple inputs single output (MMISO) algorithms and improved the detection accuracy of multiple single gas concentrations. A method combining Weighted Kernels Fisher Discriminant Analysis (WKFDA) with Quantum-behaved Particle Swarm Optimization (QPSO) and reprocessing of an original eigenmatrix using QWKFDA was proposed by Li, Z.H. et al. [36], improving the accuracy of feature parameter extraction in the prediction of wound infection and inflammable gases. Reference [37] proposed a hybrid gas detection method based on one-class support vector machines (SVM). The recognition rates of the two gas samples reached 95.24% and 94.83%, respectively.

Although the above-mentioned methods have effectively achieved mixed gas identification and concentration detection to some extent, both PCA and ICA are linear feature extraction methods [38], and the extracted features are linear additions of the original features. However, the response signals of MOS gas sensors have nonlinear characteristics [39]. The intrinsic structure of the actual data set is not in the same plane; thus, the PCA and ICA methods are not ideal. The ANN algorithm requires a large number of parameters, and the empirically determined weights and thresholds will cause the gas recognition rate to fluctuate. Moreover, the ANN algorithm requires a large number of learning processes, which may be infeasible for small sample data sets. The SVM classification model [40] used for small samples and nonlinear problems is limited by the fact that the kernel function must satisfy the Mercer condition. With an increase in the number of training samples, the number of support vectors increases linearly, and the model sparsity is greatly reduced. SVM requires parameter optimization to achieve the best recognition rate, that greatly increases the amount of computation [41].

To resolve the nonlinear characteristics of MOS gas sensor responses to mixed gas signals, we present KPCA as a feature extraction method for mixed gas signals. KPCA addresses the limitations of PCA for extracting nonlinear data features. Through kernel functions, nonlinear data in a low-dimensional space are mapped to a high-dimensional space for analysis to achieve nonlinear feature extraction [42]. The K-nearest neighbour (KNN) algorithm is used as a classification method. The algorithm has a clear and simple objective and is highly mature. The KNN algorithm can achieve a higher classification accuracy for mixed gases by using the KPCA feature extracted signal [43]. The Multivariate Relevance Vector Machine (MVRVM) is used as the concentration regression method. MVRVM was presented by Thayanantheana et al. in 2006 as a method for simultaneously regressing multi-input variables [44]. The technique is widely used in fault diagnosis [45] and geomagnetic prediction [46]. MVRVM is based on a hierarchical Bayesian probability model structure and is an extension of the correlation vector machine algorithm. The algorithm requires less sample data and offers high prediction accuracy and strong generalization ability [47]. Under the structure of a priori parameters, autocorrelation decision theory is used to remove irrelevant points and obtain a sparse model. Multiple probability probabilistic functions are introduced to achieve multiple outputs to reduce computational complexity. Hence, the complex nonlinear relationship between the mixed gas concentration and the response signal of the MOS gas sensor array can be addressed by MVRVM to realize the regression of a mixed gas concentration. The contributions of this paper are summarized as follows:

(1) This paper proposes a KPCA-KNN gas identification method aiming at the low identification rate of binary mixed gas in the existing machine olfactory system. The method uses KPCA to extract the nonlinear characteristics of a binary mixed gas with different concentration ratios, composes the mixed gas feature set, and then uses a KNN classifier to identify the gases.
(2) To improve binary mixture gas detection accuracy, this paper proposes to use MVRVM’s multi-input multi-output feature, with the MOS gas sensor array’s response signal as the input and the two target gas concentrations as the output, to achieve binary mixed gas concentration detection.
(3) The accuracy of the proposed method is verified by qualitative analysis and quantitative detection of CO and CH_4_ mixed gases. The experimental results show that the proposed method has better resolution accuracy for binary mixed signals than other methods do.

The rest of this article is organized as follows: the signal feature extraction method KPCA and the classification algorithm KNN are described in Section 2. The multiclass relevance vector machine method is introduced in Section 3. Section 4 describes the mixed gas detection method, including gas identification and concentration. Section 5 details simulation experiments based on CO and CH_4_ gas detection. Validation experiments are also presented. The major findings of this work are summarized in Section 6.

## 2. Mixed Gas Qualitative Identification

### 2.1. KPCA Feature Extraction

Kernel principal component analysis (KPCA) maps nonlinear raw data from input space to high-dimensional space ℤ through kernel function Φ(·) and then uses principal component analysis to extract data features of nonlinear raw data in high-dimensional space ℤ.

Assumption $X=[x_{1},x_{2},\ldots ,x_{M}]\in R^{N\times M}$ represents the original observation sample. M represents the dimension of each sample, N is the number of observed samples. $x_{i}\in {\mathbb{R}}^{N}$ represents the *i*-th M-dimensional observation sample. When the nonlinear mapping function Φ(·) satisfies the centralization requirement, the formula is as follows:

(1) $$\sum_{i=1}^{M}\Phi \left(x_{i}\right)=0.$$

Then, the covariance matrix of the original observation sample in the feature space can be expressed as:

(2) $$C=\frac{1}{M}\sum_{i=1}^{M}\Phi \left(x_{i}\right)\Phi {\left(x_{i}\right)}^{T}.$$

The eigenvalue solving equation of the covariance matrix C is:

(3) $$\lambda v=Cv=\frac{1}{M}\sum_{i=1}^{M}<\Phi \left(x_{i}\right),v>\Phi {\left(x_{i}\right)}^{T}.$$

λ and v represent feature values and feature vectors, respectively. Feature vector v can be linearly represented by $\Phi (x_{1}),\Phi (x_{2}),\ldots ,\Phi (x_{M})$. Therefore, $\alpha_{i}(i=1,2,\ldots ,M)$ is defined as follows:

(4) $$v=\sum_{i=1}^{M}\alpha_{i}\Phi \left(x_{i}\right).$$

By substituting Equation (4) into Equation (3), we obtain:

(5) $$\lambda \left(\Phi \left(x_{k}\right)\cdot v\right)=\Phi \left(x_{k}\right)\cdot Cv\left(k=1,2,\ldots ,M\right).$$

An M×M nuclear matrix K is defined as follows:

(6) $$K_{\mathrm{ij}}=K\left(x_{i},x_{j}\right)=\left(\Phi \left(x_{i}\right),\Phi \left(x_{j}\right)\right).$$

By combining Equations (4)–(6), the eigenvalue solving problem can be further transformed into the following:

(7) Mλα=Kα.

Mλ is the characteristic value of nuclear matrix K, and $\alpha =(\alpha_{1},\alpha_{2},\ldots ,\alpha_{N}{)}^{T}$ is the eigenvector. Equation (7) is solved in the high-dimensional space to obtain the eigenvector $\alpha_{1},\alpha_{2},\ldots \alpha_{M}$ and its corresponding eigenvalue $\lambda_{1}\geq \lambda_{2}\geq \ldots \geq \lambda_{M}$. The dimension reduction can be achieved by retaining the first p feature vectors by the cumulative contribution rate method as follows:

(8) $$r_{CCR}=\sum_{i=1}^{p}\lambda_{1}/\sum_{j=1}^{M}\lambda_{j}\times 100\%.$$

The *k*-th principal component of the new observation sample x can be obtained by mapping Φ(x) to feature vector $v_{k}$:

(9) $$t_{k}=\langle v_{k},\Phi (x)\rangle =\sum_{i=1}^{M}\alpha_{i}^{k}\langle \Phi (x_{i}),\Phi (x)\rangle ,k=1,2,\ldots ,p.$$

p is the number of kernel principal components. The contribution rates of the kernel principal components are sorted, and the first p principal components are taken such that the cumulative contribution rate $r_{CCR}$ is at least 85%.

It is worth noting that when the observed sample does not meet the requirements of Formula (1), the nuclear matrix K can be replaced by the following:

(10) $$\tilde{K}=K-I_{M}K-KI_{M}+I_{M}KI_{M},$$

where $I_{M}$ is an M×M matrix:

(11) $${\left(I_{M}\right)}_{ij}=\frac{1}{M}.$$

### 2.2. KNN Proximity Algorithm

The K-nearest neighbour algorithm calculates the distance between a sample to be classified and a training sample of a known class and obtains the K training samples closest to the sample to be classified. If the K samples closest to the sample to be classified belong to the same category, then the sample to be classified also belongs to this category. If the K samples closest to the sample to be classified do not belong to the same category, it is determined that the sample to be classified belongs to the category with the highest number of K samples.

The simplest case is that in which K = 1, wherein the resulting training sample point is the closest training sample to the input sample. The hypothetical training sample is $\left\{y_{j}{}^{\left(i\right)}\right\},(i=1,2,\ldots ,c),(j=1,2,\ldots ,N_{i})$, where i denotes the sample class and j denotes the sample number in the *i*-th class. The total number of training samples is $N=\sum_{i=1}^{c}N_{i}$, where c is the total number of categories and $N_{i}$ is the number of samples of type $\omega_{i}$. The distance between sample x to be categorized and sample $y_{j}{}^{\left(i\right)}$ of the N known classes is $d_{j}{}^{\left(i\right)}$, which is determined as the class to which the sample whose $d_{j}{}^{\left(i\right)}$ is the smallest belongs. The decision function is expressed as follows:

(12) $$d_{i}\left(x\right)=\underset{j=1,2,\ldots ,N_{i}}{\min}\left\|x-y_{j}^{\left(i\right)}\right\|,i=1,2,\ldots ,c.$$

The rules of judgement are expressed as:

(13) $$x\in \omega_{m},m=\arg\underset{i=1,2,\ldots ,c}{\min}\left(d_{i}\left(x\right)\right).$$

1NN uses the nearest training sample as the determination condition. Obviously, this is a simple, intuitive method of classification. However, when the number of samples in the training dataset is large, the method of using this distance from a single sample as a classification criterion has a certain probability of producing an error, resulting in a low classification accuracy. To improve the accuracy of the classification, the number of training samples examined is extended to k nearest neighbours. KNN is an extension of the 1NN method. In the training sample set, the nearest k neighbours of the input sample are found, and then the decision rule is used to determine the category of the input sample.

Let $k_{1},k_{2},\ldots ,k_{c}$ be the number of k nearest neighbours for x. The categories are $\omega_{1},\omega_{2},\ldots \omega_{c}$, and the decision function $\omega_{i}$ is:

(14) $$d_{i}\left(x\right)=k_{i},i=1,2,\ldots c.$$

The judgement rule is:

(15) $$x\in \omega_{m},m=\underset{i=1,2,\ldots ,c}{\max}\left(d_{i}\left(x\right)\right).$$

When designing the nearest neighbour classifier, a metric function is needed to measure the distance between samples, which gives the size of the scalar distance between two samples. Euclidean distance is the most common distance metric function. In the supervised classification problem, two samples containing l attributes are defined as Euclidean distances between $Χ=(x_{1},x_{2},\cdots ,x_{l})$ and $Ζ=(x_{1},x_{2},\cdots ,x_{l})$:

(16) $$D_{E}(Χ,Ζ)=\sqrt{\sum_{i=1}^{l}{\left(x_{i}-z_{i}\right)}^{2}}.$$

Although the Euclidean distance formula can always be used to calculate the distance between two vectors, the resulting distance value is not always meaningful. For example, if the coordinates are transformed and each coordinate axis is multiplied by an arbitrary constant, the actual transformation of this coordinate simply changes the unit of each attribute. However, the relationship between the Euclidean distance in the transformed space and the distance in the original space may be completely different.

The metric in the more general d space is the Minkowski distance metric. For two points X and Z in d space, the Minkowski distance between them is calculated as follows:

(17) $$L_{k}(Χ,Ζ)=\left(\sum_{i=1}^{d}|x_{i}-z_{i}{|}^{k}\right){}^{1/k}.$$

Such a distance metric is also called the $L_{k}$ norm, and the Euclidean distance is the $L_{2}$ norm. The $L_{1}$ norm is the Manhattan distance, where $L_{1}(Χ,Ζ)$ represents each segment of the nearest distance from the X point to the Z point that is parallel to the corresponding coordinate axis. The $L_{\infty }(Χ,Ζ)$ norm represents the maximum value among the distances between the projection of the X point and the Z point to the d coordinate axes.

## 3. Mixture Gas Concentration Estimation

The Multivariable Relevance Vector Machine (MVRVM) is a supplement and extension to the Relevance Vector Machine (RVM), which can realize the simultaneous regression of multiple variables. MVRVM still exhibits good generalization ability under small-sample conditions and can guarantee the accuracy of regression. The model is sparse, and the complexity is not high, which is conducive to confirming the real-time output of the measured value. As a kernel learning method, MVRVM maps the complicated input-output relationship of a gas sensor to a linear high-dimensional space, which can help solve the corresponding nonlinear problem. This method is suitable for solving concentration estimation problems based on MOS gas sensor arrays.

Given training sample set ${\left[x^{\left(n\right)},t^{\left(n\right)}\right]}_{n=1}^{N}$, $x^{\left(n\right)}\in R^{1\times q}$ and $t^{\left(n\right)}\in R^{1\times m}$ represent the multi-dimensional response signal and target gas concentration vector of the nth MOS gas sensor array, q is the number of gas sensor installed in the MOS gas sensor array, M is the number of different gas types in the mixed gas, and E is the number of training samples. The mathematical expression of the multiple regression model based on MVRVM is as follows:

(18) $$y^{\left(n\right)}=\Phi \left[x^{\left(n\right)}\right]\cdot W,$$

where $y^{\left(n\right)}\in R^{1\times M}$ is the output value of the MVRVM regression model, i.e., the predicted value output vector of the *n*th set of sample data sets. $y^{\left(n\right)}=\left[y_{1},y_{2},\cdots y_{m,\cdots }y_{M}\right]$, 1≤m≤M, M is the number of outputs.

$\bar{W}\in R^{RV\times M}$ is the weight matrix optimized by the regression model $\bar{W}=\left[{\bar{w}}_{1}{\bar{w}}_{2}\cdots {\bar{w}}_{m}\cdots {\bar{w}}_{M}\right]$, ${\bar{w}}_{m}={\left[{\bar{w}}_{m1}{\bar{w}}_{m2}\;\cdots {\bar{w}}_{mrv}\;\cdots {\bar{w}}_{mRV}\right]}^{T}$, 1≤rv≤RV. RV is the number of correlation vectors selected from the N training samples in the MVRVM model, and RV≪N; $\bar{\Phi }\left[x^{\left(n\right)}\right]\in R^{l\times RV}$ is the optimal design matrix, which is the kernel mapping matrix of the first set of sample data sets, where $\bar{\Phi }=K\left\{x^{\left(n\right)},{\left[x^{\left(*\right)}\right]}_{rv=1}^{RV}\right\}$, K(⋅), $x^{\left(*\right)}$, and RV denote the kernel function matrix, kernel function, correlation vector and the number of correlation vectors, respectively.

The solution process of the multiple regression model based on MVRVM is as follows:

Assume that the weight matrix W obeys the prior normal distribution, as indicated in Equation (19), and the likelihood distribution of the weight matrix W is as indicated in Equation (20):

(19) $$p\left(W|A\right)=\prod_{m=1}^{M}\prod_{n=1}^{N}N\left(\omega_{mn}|0,\alpha_{n}^{-2}\right),$$

(20) $$p\left({\left\{t^{\left(n\right)}\right\}}_{n=1}^{N}|W,B\right)=\prod_{m=1}^{M}\left(t_{m}|W\cdot \Phi ,B\right),$$

(21) $$\Phi =K\left\{{\left[x^{\left(n\right)}\right]}_{n=1}^{N},{\left[x^{\left(n\right)}\right]}_{n=1}^{N}\right\},$$

(22) $$A=diag\left(\alpha_{1}{}^{-2},\alpha_{2}{}^{-2},\cdots \alpha_{n}{}^{-2},\cdots ,\alpha_{N}{}^{-2}\right),$$

(23) $$B=diag\left(\beta_{1},\beta_{2},\ldots ,\beta_{m},\ldots ,\beta_{M}\right).$$

In (22), the element $\alpha_{n}$ is called the hyperparameter of the correlation vector and is used to select the training samples that make up the correlation vector. In Equation (23), $\beta_{m}$ denotes the noise signal included in the *m*-th estimated output, and $\omega_{mn}$ denotes the element in the *m*-th row and the *n-*th column in the weight matrix W.

The prior probability distribution of the weight matrix W is indicated in Equation (24). The posterior probability distribution of W is the inner product of each weight vector that is independent and obeys the Gaussian distribution, as indicated in Equation (25). Further deduction yields Equation (26):

(24) $$p\left(W|A\right)=\prod_{m=1}^{M}N\left(\omega_{m}|0,A\right),$$

(25) $$p\left(W{|\left\{t^{\left(n\right)}\right\}}_{n=1}^{N},B,A\right)\propto \left({\left\{t^{\left(n\right)}\right\}}_{n=1}^{N}|W,B\right)\cdot p(W,A)\;$$

(26) $$p\left(W{|\left\{t^{\left(n\right)}\right\}}_{n=1}^{N},B,A\right)\propto \prod_{m=1}^{M}N(w_{m}|\mu_{m},\sum_{m}),$$

where $\mu_{m}=\beta_{m}^{-1}\sum_{m}\Phi^{T}\tau_{m}$ is the mean of the weight matrix and $\sum_{m}={\left(\beta_{m}^{-1}\Phi^{T}\Phi +A\right)}^{-1}$ is the variance vector.

Finally, by maximizing the maximum edge likelihood function of the objective function, the optimal hyperparameter and noise parameters are obtained, as indicated in Equations (27) and (28):

(27) $$\bar{A}=diag\left({\bar{\alpha }}_{1}^{2}\;{\bar{\alpha }}_{2}^{2}\cdots {\bar{\alpha }}_{rv}^{2}\cdots {\bar{\alpha }}_{RV}^{2}\right),$$

(28) $$\bar{B}=diag\left({\bar{\beta }}_{1}\;{\bar{\beta }}_{2}\cdots {\bar{\beta }}_{m}\cdots {\bar{\beta }}_{M}\right)\;$$

The resulting averaged vector ${\bar{\mu }}_{m}\in R^{RV\times 1}$ and weight matrix $\bar{W}\in R^{RV\times M}$ expression are

(29) $${\bar{\mu }}_{m}={\bar{\beta }}_{m}{\bar{\sum }}_{m}{\bar{\Phi }}^{T}\tau_{m},$$

(30) $$\bar{W}={\left({\bar{\mu }}_{1},\cdots {\bar{\mu }}_{M}\right)}^{T}.$$

The optimized variance matrix $\sum_{m}\in R^{RV\times RV}$ in Equation (29) is expressed as follows:

(31) $${\bar{\sum }}_{m}={\left({\bar{\beta }}_{m}^{-1}{\bar{\Phi }}^{T}\bar{\Phi }+\bar{A}\right)}^{-1}.$$

Correspondingly, if the latest test sample is denoted as $x_{*}\in R^{p\times q}$, p is the number of test samples, q is the number of MOS gas sensors, and $y_{*}\in R^{p\times M}$ is the output value of the multiple regression model based on MVRVM:

(32) $$y_{*}=\bar{\Phi }{\left[x_{*}\right]}_{p\times RV}\cdot {\bar{W}}_{RV\times M}\;$$

The error vector is represented by the diagonal elements of the matrix $\sigma_{y}$ and is expressed as follows:

(33) $$\sigma_{y}=sprt\left({\bar{B}}^{-1}+\bar{\Phi }\cdot \;\bar{\Sigma }\cdot {\bar{\Phi }}^{T}\right).$$

When calculating the optimal hyperparameters, as the number of iterations increases, many hyperparameters will tend to infinity. As most of the corresponding weights tend to zero, an increasing number of sample vectors in the training data set will be rejected, and fewer correlation vectors will be preserved, thus sparsifying the model.

## 4. Hybrid Gas Detection Method

**Figure 2:**
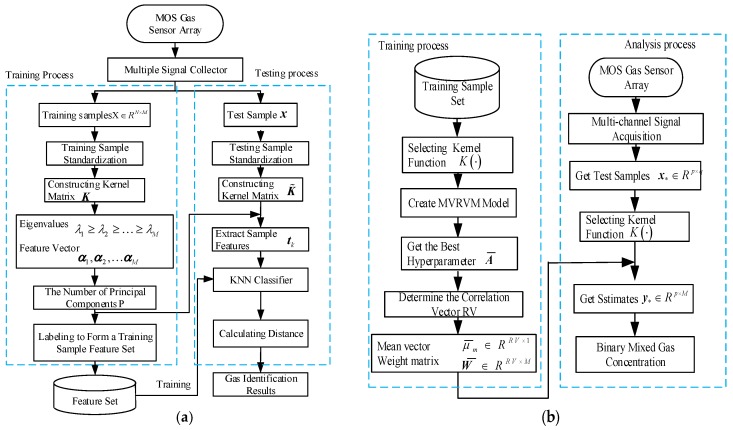
(**a**) Flow chart of binary mixed gas identification method based on KPCA and KNN; (**b**) flow chart of binary mixed gas concentration estimation method based on MVRVM.

KPCA possess a powerful ability to extract useful features from nonlinear signals, mapping the extracted feature data into a space that facilitates classification. In the classification algorithm, KNN algorithm offers higher accuracy and lower training time complexity. Therefore, this paper proposes a new hybrid gas type identification method based on the KPCA and KNN algorithms. A flow chart of the proposed method is presented in Figure 2a, and is illustrated in the following steps.

Step 1: Use the MOS gas sensor array to collect the response signals of mixed gas samples of different compositions. To remove the influence of the baseline, subject the collected data to a baseline reduction process.
Step 2: By constructing a kernel matrix K from the training sample set, use KPCA to extract the features of all training samples and forms a training sample feature set.
Step 3: Use the feature vector of the training sample set obtained by KPCA to obtain the characteristics of the test sample.
Step 4: Identify the characteristics of the test sample using the KNN algorithm, select the K points with the smallest distance, and count the number of occurrences of the category to which the K-point belongs the most. The category corresponding to the most frequent point is the category of the measured point.

For the gas concentration estimation problem, the Multivariate Relevance Vector Machine (MVRVM) has the characteristics of strong generalization ability for small sample data, high regression accuracy, and sparse model. In this paper, a new mixed gas concentration estimation method is proposed based on MVRVM. A flow chart of the proposed method is presented in Figure 2b:

Step 1: Collect the response signals of the mixed gas samples with different concentrations through the MOS gas sensor array. To remove the influence brought by the baseline, subtract the baseline data from the collected data signals.
Step 2: For the training sample set, select the kernel function K, establish the relevant MVRVM model, obtain the optimal hyperparameter, and determine the number of related vectors to obtain the mean vector and the weight matrix.
Step 3: Calculate the estimated gas concentration by calculating the mean value vector and the weight matrix.

## 5. Experiment

### 5.1. Experimental Sample Acquisition

**Figure 3:**
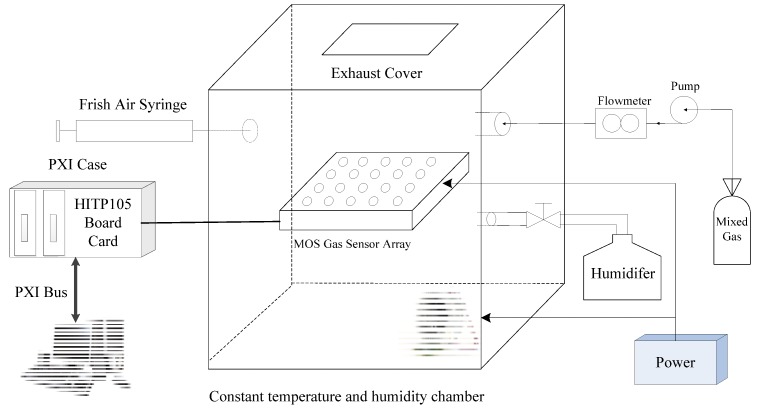
Binary mixed gas detection experimental system.

To verify the feasibility and effectiveness of the binary mixed gas detection method in the machine olfactory system, a binary gas detection experiment system was designed to analyse the performance of the method. The experimental system block diagram is presented in Figure 3.

The experimental system is mainly composed of a gas sensor array, an AD acquisition board, a PXI chassis, a host computer, and a DC power supply. The MOS gas sensor array consists of five Figaro sensors with different sensitivities: TGS2600, TGS2610, TGS2611, TGS2602, and TGS2620. To improve the reliability of the sensor array under experimental conditions, five types of gas sensors are selected for each type to form a 5 × 4 array. The array consisting of multiple sensors has a certain universality. Each sensor selects four, which ensures that the sensor array has a certain fault-tolerant ability. When one or several sensors have problems, other sensors can be used instead. On the other hand, it can eliminate the problem that the same sensor has different response to gas due to manufacturing process problems. The AD acquisition board uses an independently designed P105 function board, and this board is based on the acquisition function of DSP and FPGA, with 32 channels. In this project, only the first 20 channels are used to collect the voltage signal output by the sensor in real time, and the signal is saved in the txt file. The sampling rate of the board is set to 10 Hz, the input range of the signal is −9 V~+9 V, the A/D resolution is 16 bits, and the full-scale accuracy is 0.5%. The AD acquisition board uses the CPCI interface to communicate with the host computer. The PXI chassis uses a PXI-1042 produced by NI Corporation. The DC power supply provides a +5 V supply voltage and heating voltage. Because the response characteristics of the sensor array are susceptible to temperature and humidity, the selected sensor has the best gas selectivity at 15 °C and relative humidity of 20%. Therefore, signal acquisition must be performed in a room with constant temperature and humidity, where the constant temperature and humidity are ensured by a fan and a humidifier [48]. The experimental conditions are set to 15 °C and a relative humidity of 20%. The procedure for obtaining the experimental sample is as follows: the binary mixed gas (CH_4_ and CO) is prepared, and mixed gas of various concentrations is injected into the gas chamber. Before each different concentration of gas is injected into the gas chamber, 300 s of pure air is injected first, and then the next concentration of mixed gas is injected. The response output value of the sensor array is recorded as an experimental sample.

### 5.2. Experimental Sample Composition

Table 1 presents the sample composition of the mixed gas test, with 50 different mixed concentration combinations. TS represents the training sample, ES represents the test sample, and each concentration is sampled five times. Each mixed gas sample is continuously collected for 1 s at a sampling frequency of 10 Hz after the sensors signal reached a stable value such that the data obtained each time form a 10 × 20 matrix. To ensure the reliability of the data, five acquisitions were performed such that the data size for each concentration was 50 × 20. There were 26 training samples and 24 test samples. Therefore, the dimensions of the training sample matrix were 1300 × 20, and those of the test sample matrix were 1200 × 20.

### 5.3. MOS Gas Sensor Sensitivity Analysis

The detection principle of the target gas by the MOS gas sensor is such that, at a certain heating temperature, the surface of the gas sensor can adsorb oxygen molecules O in the environment. Oxygen molecules obtain electrons from the surface of the gas-sensitive material and form charged particles O^−^ and O^2−^, etc., resulting in a decrease in the number of electrons on the surface of the material and an increase in the surface resistance of the gas-sensitive material. When reducing gases (CH_4_, CO) occur in the environment, the oxidation-reduction reaction on the surface of the semiconductor material will cause the electrons in O^−^ and O^2−^ to return to the semiconductor material, causing the surface resistance value of the gas sensitive material to decrease. It can be observed that the sensitivity of MOS gas sensors is based on complex physical and chemical reactions. The MOS gas sensor detects the target gas through the abovementioned process. The chemical reaction equations of the detection principle are presented as Equations (34)–(36):

(34) $$\frac{1}{2}O_{2}+ne\to O_{\mathrm{Adsorption}}^{n-},$$

(35) $$O_{\mathrm{Adsorption}}^{n-}{+\mathrm{CH}}_{4}\to H_{2}O+{\mathrm{CO}}_{2}+ne,$$

(36) $$O_{\mathrm{Adsorption}}^{n-}+\mathrm{CO}\to {\mathrm{CO}}_{2}+ne.$$

**Figure 4:**
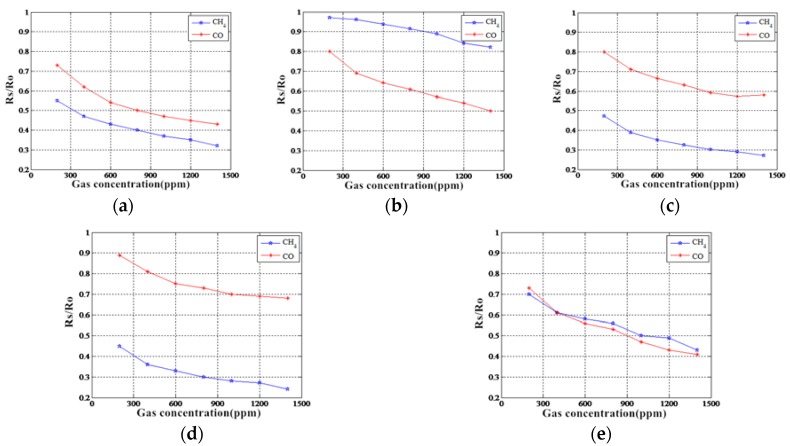
Sensitivity characteristic curves of MOS gas sensor array response to different target gases. (**a**) TGS2600 sensitivity characteristic; (**b**)TGS2602 sensitivity characteristic; (**c**) TGS2610 sensitivity characteristic; (**d**) TGS2611 sensitivity characteristic; (**e**) TGS2620 sensitivity characteristic.

To investigate the sensitivity to methane gas and carbon monoxide gas of the MOS gas sensors produced by the five commercially available Figaro companies, this paper analyses the sensitivity characteristics of each sensor under different concentrations of methane gas and carbon monoxide gas. The MOS gas sensor array’s sensitivity curve corresponding to the different target gases is presented in Figure 4. The MOS gas-sensitive materials demonstrate different sensitivity characteristics for different target gases; specifically, they exhibit nonlinear changes in sensitivity to the same target gas concentration, and their selectivity is not singular, i.e., there are cross-sensitivity characteristics.

**Figure 5:**
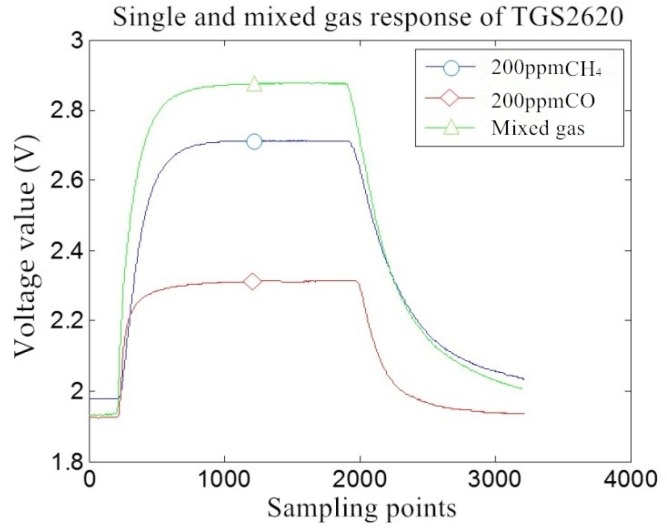
The response process curve of a single and mixed gas of the TGS2620 sensor.

As presented in Figure 5, the single and mixed gas response curve of the TGS2620 sensor is taken as an example. The sensor shows cross-sensitivity characteristics for methane gas and carbon monoxide gas. The response output of the sensor to the mixed gas is not equal to the sum of the responses of the sensor to the two target gases and has a nonlinear characteristic.

Therefore, regarding the characteristics of the MOS gas-sensitive materials, it is not possible to obtain exact information about the target gas species or concentration through the sensor output. The incorporation of the subsequent signal processing method can reveal the target gas information via its response signal and then select an appropriate pattern recognition method for analysis.

## 6. Binary Gas Detection

The kernel function in the KPCA algorithm employed the most commonly used Gaussian radial kernel function $K\left(x_{i},x_{j}\right)=\exp\left(-{\left\|x_{i}-x_{j}\right\|}^{2}/\sigma^{2}\right)$. The choice of kernel parameters was based on cross-validation method and was ultimately determined to be σ=2.5. As shown in Table 2, when the cumulative contribution rate of the principal component reached 95%, the principal component reached 43; that is, the number of dimensions of the data was increased from the original 20 to 43. After KPCA processing, the dimensions of the training data set and test data set were 1300 × 43 and 1200 × 43, respectively. In the KNN classifier, the value of k was determined to be 5 after several experiments. After feature extraction, the data were incorporated into the KNN classification model to obtain the final recognition rate. Table 3 shows the three feature extraction methods for CO, CH_4_, mixed gas, and average recognition rate.

Table 3 shows that the average recognition rate obtained by the KPCA method is 5.83% and 14.16% higher than the rates of PCA and ICA, respectively, reaching 98.33%. Therefore, the proposed method can extract feature information about the multi-dimensional response signal of the MOS sensor array better, thereby improving the recognition rate of the binary mixed gas species.

The quantitative analysis of the mixed gas concentration was based on the results of a qualitative analysis. The gas concentrations were estimated using the MVRVM method for a single gas and a mixed gas, respectively. The kernel functions all employed the most commonly used Gaussian radial basis function, and the optimal kernel parameters were solved by a 5-fold cross-validation method. The binary mixed gas concentration estimation results are shown in Table 4.

For single gas predictions, the optimal kernel parameters for CO and CH_4_ were 0.76 and 0.25, and the average relative errors were 2.36% and 2.01%, respectively. The prediction result for mixed gas was an optimal kernel parameter value of 0.67, and the average relative errors of CO and CH_4_ were 9.01% and 8.79%, respectively.

To illustrate the performance of the MVRVM binary mixed concentration detection method proposed in this paper, Table 5 compares the binary mixture gas concentration detection performances of different methods. The table shows that the MVRVM binary mixed gas concentration detection method offers a lower average relative error than does the single RVM method or the LS-SVR method, and the average detection time is significantly reduced.

## 7. Conclusions

Based on the metal oxide gas sensor array, the detection accuracy of mixed gas in the machine olfactory system is low. This paper proposes a feature extraction method based on KPCA. Combined with the binary mixed gas identification model of the KNN classification algorithm, qualitative identification of mixed gas is realized. For the qualitative identification results, a regression method based on MVRVM was proposed to achieve quantitative detection of gas concentration. The major findings of this work can be summarized as follows:

(1) KPCA was verified as a feature extraction method for processing nonlinear signals. Compared with PCA and ICA, KPCA exhibits a good signal feature extraction capability. Using the KNN classification algorithm to construct a gas identification model, the recognition accuracy rate exceeds 98%.
(2) This study also examined the detection of mixed gas concentrations and proposed an MVRVM algorithm that is different from the ANN and requires many training cycles. The average relative error of gas concentration monitoring is within 6%, and the detection time is short, which is more suitable than other methods for real-time detection of mixed gas.
(3) The method for qualitative identification and quantitative detection of the binary mixed gas proposed in this paper was verified via experiments, and the accuracy of detection and the detection of a mixed gas by the machine olfactory system was improved. It is worth expanding the application of the system to the identification and detection of multiple gas mixtures.

## Figures and Tables

**Table 1:** Experimental sample composition.

CH_4_ (ppm)	CO (ppm)							
	0	200	400	600	800	1000	1200	1400
0		TS	ES	TS	ES	TS	ES	TS
200	TS	ES	TS	ES	TS	ES	TS	
400	ES	TS	ES	TS	ES	TS	ES	
600	TS	ES	TS	ES	TS	ES	TS	
800	ES	TS	ES	TS	ES	TS	ES	
1000	TS	ES	TS	ES	TS	ES	TS	
1200	ES	TS	ES	TS	ES	TS	ES	
1400	TS							

**Table 2:** KPCA characteristic value and contribution rate.

Principal Component	Eigenvalues	Contribution Rate	Cumulative Contribution Rate
PC1	0.1072	11.96%	11.96%
PC2	0.0932	10.40%	22.36%
PC3	0.0739	8.25%	30.61%
PC4	0.0565	6.30%	36.91%
PC5	0.0524	5.85%	42.76%
PC6	0.0432	4.82%	47.58%
PC7	0.0373	4.17%	51.75%
…	…	…	…
PC32	0.0055	0.60%	90.31%
…	…	…	…
PC43	0.0027	0.29%	95.11%

**Table 3:** Recognition results corresponding to PCA, ICA and KPCA.

Category	Sample	Detection Sample Recognition Rate		
		PCA	ICA	KPCA
CO	150	86.70%	100%	93.30%
CH_4_	150	100%	53.30%	100%
Mixed Gas	900	92.20%	86.70%	98.80%
Average	-----	92.5%	84.17%	98.33%

**Table 4:** Binary mixed gas concentration estimation results.

Gas Category	Single Gas		Mixed Gas	
Gas Composition	CO	CH_4_	CO	CH_4_
Optimal Kernel Parameters	0.76	0.25	0.67	
Average Relative Error	2.36%	2.01%	9.01%	8.79%

**Table 5:** Comparison of binary mixed gas concentration detection performance.

Performance	Method		
	MVRVM	Single RVM	LS-SVR
Average Relative Error of CO (%)	5.58	6.16	7.85
Average Relative Error of CH_4_ (%)	5.38	7.17	5.65
Average Detection Time (ms)	1.37	22.86	91.63
